# Characterization of the *Acinetobacter baumannii* Secretome Using Size-Exclusion Chromatography and Raman Spectroscopy

**DOI:** 10.3390/ijms27135904

**Published:** 2026-06-30

**Authors:** Elizaveta Alekseevna Denisova, Anastasia Avdyusheva, Elizaveta Tyshchuk, Polina Grebenkina, Andrey Korenevsky, Ivan Chelibanov, Vladimir Chelibanov, Areg Totolian, Lyudmila Kraeva, Vitaly Nazarov, Dmitry Sokolov

**Affiliations:** 1 Saint-Petersburg Pasteur Institute, St. Petersburg 197101, Russia; liza.denisova9898@yandex.ru (E.A.D.); lisatyshchuk@yandex.ru (E.T.); grebenkinap@gmail.com (P.G.); a.korenevsky@yandex.ru (A.K.); ichelibanov@gmail.com (I.C.); totolian@pasteurorg.ru (A.T.); lykraeva@yandex.ru (L.K.); 2 Research Institute of Obstetrics and Gynecology Named After D.O. Ott, St. Petersburg 199034, Russia; avdyusheva04@mail.ru; 3 «OPTEC» Joint Stock Company, St. Petersburg 199178, Russia; chelibanov@gmail.com; 4 Department of Microbiology, Military Medical Academy Named After S.M. Kirov, 6 Akademika Lebedeva Street, Building 6, St. Petersburg 194044, Russia; 5 Department of Faculty Surgery, North-Western State Medical University Named After I. I. Mechnikov, 41 Kirochnaya Street, St. Petersburg 191015, Russia; venazarov@yandex.ru

**Keywords:** *Acinetobacter baumannii*, ESKAPE pathogens, bacterial secretome, Raman spectroscopy, size-exclusion chromatography, metabolite profiling, virulence factors

## Abstract

*Acinetobacter baumannii*, a multidrug-resistant pathogen of critical priority within the ESKAPE group, poses a significant threat to global healthcare, particularly in the context of nosocomial infections. Its pathogenesis is mediated not only by antibiotic resistance determinants but also by a complex repertoire of secreted virulence factors. However, comprehensive characterization of the *A. baumannii* secretome remains methodologically challenging due to spectral overlap in complex biological matrices. In this study, we applied a hybrid approach integrating size-exclusion chromatography with Raman spectroscopy to deconvolute the cell-free supernatant of *A. baumannii*. Chromatographic fractionation into seven fractions reduced spectral complexity and enabled the identification of unique metabolic profiles. Fraction 3 exhibited a distinct composition, containing specific markers for phosphatidylserine (~1724 cm^−1^), cysteine, phosphatidylinositol, and DNA (~770–806 cm^−1^), as well as CH_2_ groups of lipids and amino acids (~1450–1456 cm^−1^), while lacking signals corresponding to methionine-containing compounds, nucleic acid backbones, and polypeptide backbones characteristic of other fractions. Analysis revealed distinct biochemical specialization across fractions: Fraction 2 was enriched in glutamine/asparagine-associated signals (~990, ~998 cm^−1^), Fraction 4 contained a unique carotenoid marker (~1154 cm^−1^), Fraction 6 exhibited a phenylalanine-specific peak (~1104 cm^−1^), and Fraction 7 demonstrated the highest intensity of cysteine-containing protein, nucleotide, and phospholipid signals. These findings open new avenues for the discovery of biomarkers associated with virulence and antibiotic resistance in *A. baumannii*.

## 1. Introduction

Infections caused by multidrug-resistant (MDR) microorganisms represent a serious threat to global public health in the 21st century. Pathogens of the ESKAPE group (*Enterococcus faecium*, *Staphylococcus aureus*, *Klebsiella pneumoniae*, *Acinetobacter baumannii*, *Pseudomonas aeruginosa*, and *Enterobacter* spp.) predominate in nosocomial infections due to their ability to rapidly acquire resistance genes, form biofilms, and persist in hospital settings [1].

*Acinetobacter baumannii* is a Gram-negative coccobacillus and one of the leading causes of healthcare-associated infections, particularly in intensive care units. This opportunistic pathogen is responsible for a wide spectrum of severe nosocomial infections, including skin and soft tissue infections, wound infections, urinary tract infections, ventilator-associated pneumonia, bacteremia, and secondary meningitis. Infections most frequently develop in patients with serious comorbidities, immunosuppression, or following complex surgical interventions [2].

The global threat posed by this pathogen is associated with its exceptional capacity to rapidly acquire resistance genes, resulting in the emergence of multidrug-resistant (MDR) strains [3]. Many *A. baumannii* strains exhibit extensive resistance to multiple antibiotic classes, including ampicillin-sulbactam, carbapenems, aminoglycosides, tetracyclines, and fluoroquinolones [4]. Of particular concern is the emergence of pan-resistant isolates demonstrating resistance to all major antibacterial agents, including colistin, polymyxin B, and tigecycline. Infections caused by resistant *A. baumannii* strains are estimated to significantly increase mortality rates, prolong hospitalization duration, and elevate healthcare costs [3,4].

The pathogenesis of *A. baumannii* infections is driven not only by genetic determinants of antibiotic resistance but also by a complex repertoire of secreted virulence factors [3,4]. The cell-free supernatant (secretome) contains hundreds of bioactive molecules—including siderophores, capsular polysaccharides, enzymes, outer membrane vesicles, peptides, and metabolites—that modulate host immune responses, promote adhesion and invasion, and establish an immunosuppressive microenvironment. *A. baumannii* produces diverse exopolysaccharides and enzymes involved in stress adaptation and biofilm formation. Its secretome can suppress the oxidative burst in phagocytes, inhibit apoptosis of infected cells, and disrupt epithelial barrier integrity, thereby facilitating systemic dissemination [5,6,7].

Despite its clinical significance, the secretome of *A. baumannii*, particularly its non-proteinaceous metabolites, remains poorly characterized. Analyzing such a chemically complex mixture poses significant methodological challenges, as conventional analytical platforms often lack the selectivity and sensitivity required to detect low-abundance components within complex biological matrices [8].

Thus, comprehensive molecular characterization of the *A. baumannii* secretome constitutes a critical interdisciplinary endeavor. Elucidating the mechanisms by which *A. baumannii* metabolites interact with the host immune system is essential for developing novel strategies to combat antibiotic-resistant infections, such as targeting key virulence factors or implementing immunomodulatory interventions.

To overcome the limitations of Raman spectroscopy in analyzing complex biological mixtures—primarily spectral overlap—a hybrid approach combining chromatographic fractionation to reduce sample complexity with subsequent Raman detection has been proposed [9,10,11]. In this study, we applied this integrated strategy to analyze the secretome of *A. baumannii*, a clinically relevant ESKAPE pathogen, with the aim of identifying functionally significant fractions that may harbor key virulence or signaling molecules.

## 2. Results

**Figure A1:**
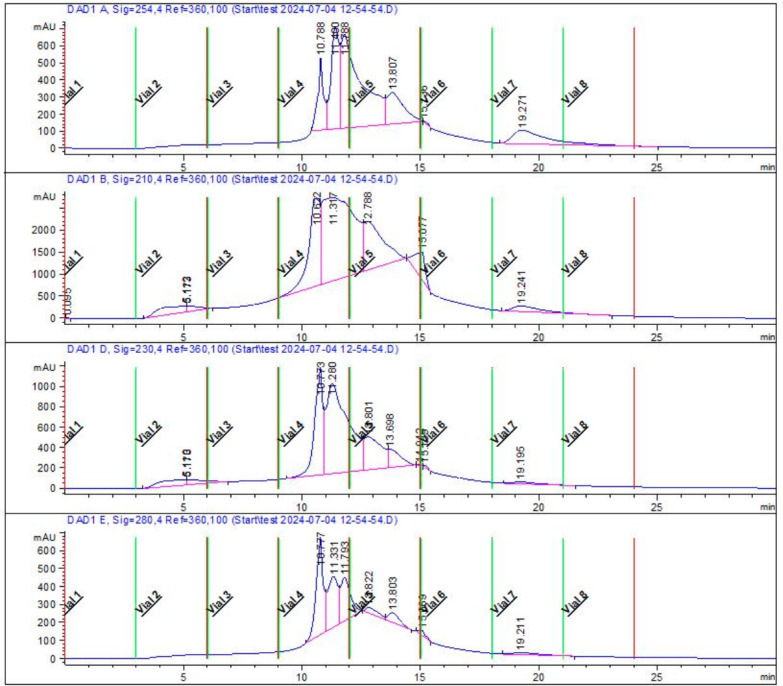
DAD detector chromatograms at wavelengths of 254.4 nm (A), 210.4 nm (B), 230.4 nm (D), and 280.4 nm (E), obtained during preparative separation of protein fractions from bacterial supernatants by gel filtration on a Bio SEC-3 column (Agilent). Peak retention times are indicated above the corresponding peaks. Samples: Vial 1—concentrated input supernatant; Vials 2–8—sequential fractions collected in time-line mode at 3-min intervals (Fractions 1–7, respectively). Analysis was performed under isocratic conditions at 4 °C, with a flow rate of 0.35 mL/min and distilled water as the mobile phase. Fractions collected after 24 min contained no detectable protein and were not further analyzed.

**Figure A2:**
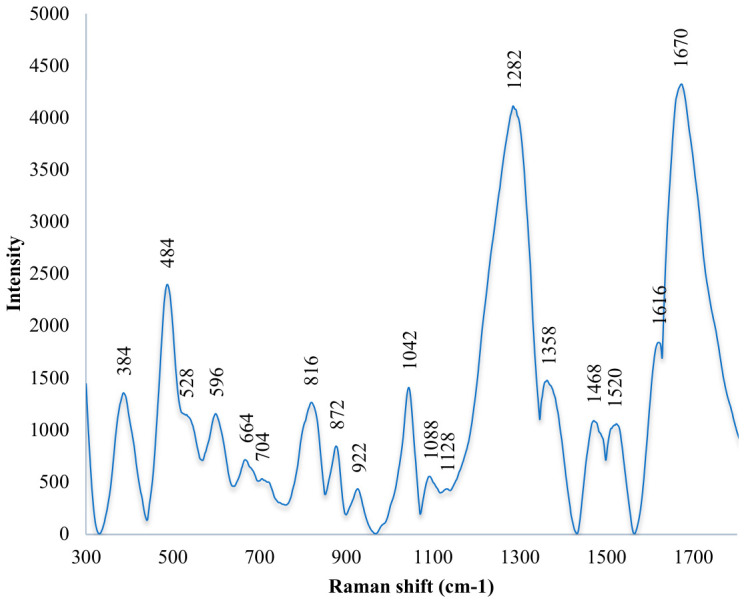
Raman spectrum of the whole cell-free supernatant of *Acinetobacter baumannii* in the range of 300–3400 cm^−1^.

**Figure A3:**
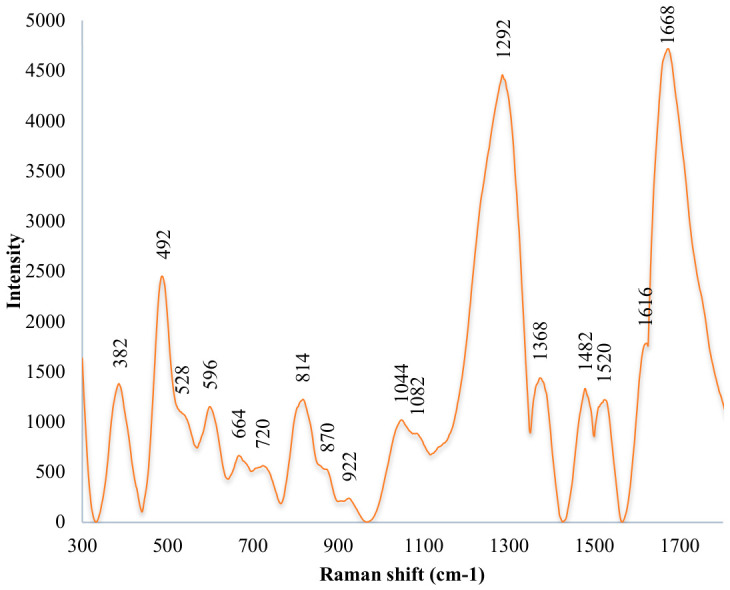
Raman spectrum of Fraction 1 of *Acinetobacter baumannii* in the range of 300–3400 cm^−1^.

**Figure A4:**
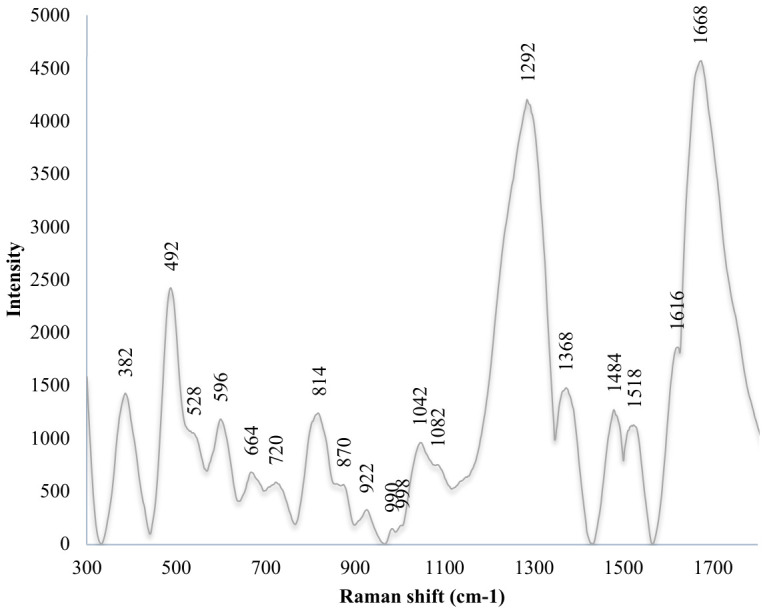
Raman spectrum of Fraction 2 of *Acinetobacter baumannii* in the range of 300–3400 cm^−1^.

**Figure A5:**
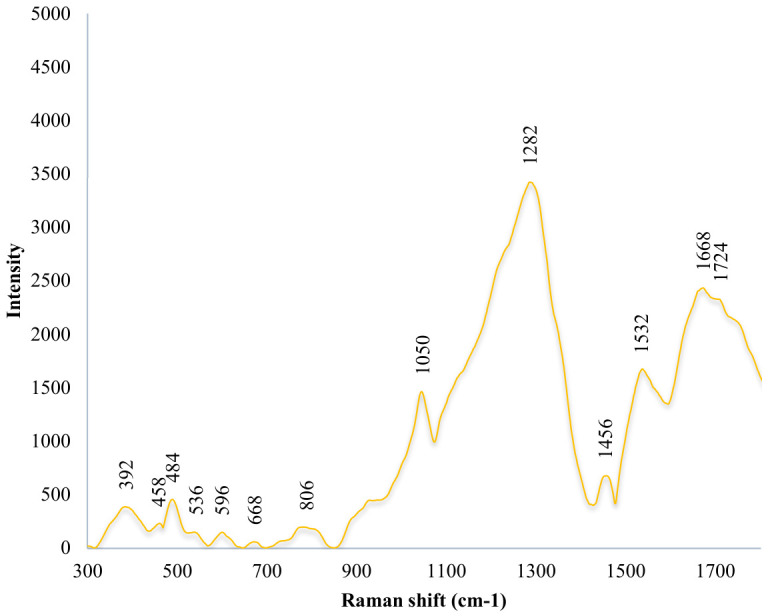
Raman spectrum of Fraction 3 of *Acinetobacter baumannii* in the range of 300–3400 cm^−1^.

**Figure A6:**
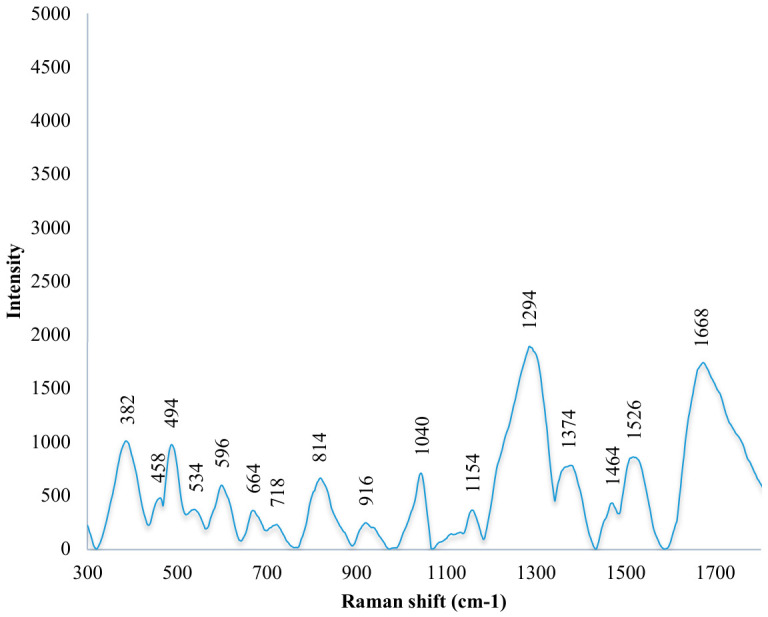
Raman spectrum of Fraction 4 of *Acinetobacter baumannii* in the range of 300–3400 cm^−1^.

**Figure A7:**
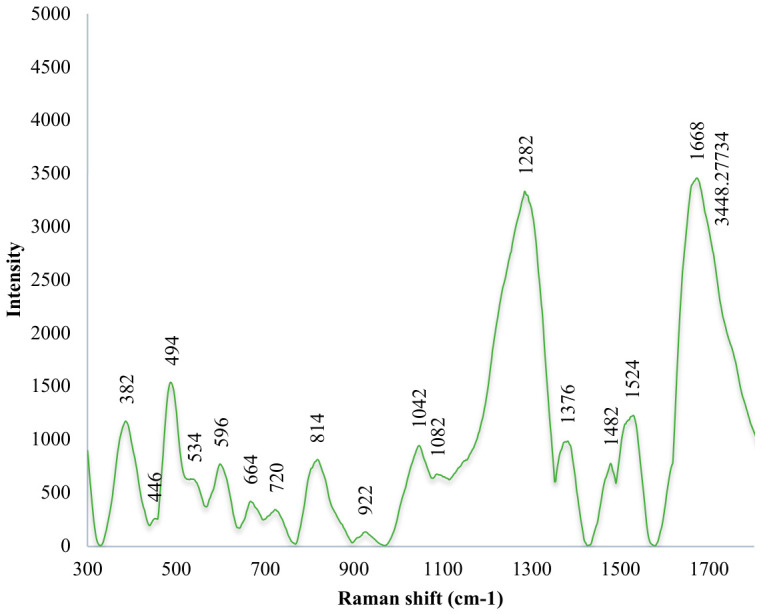
Raman spectrum of Fraction 5 of *Acinetobacter baumannii* in the range of 300–3400 cm^−1^.

**Figure A8:**
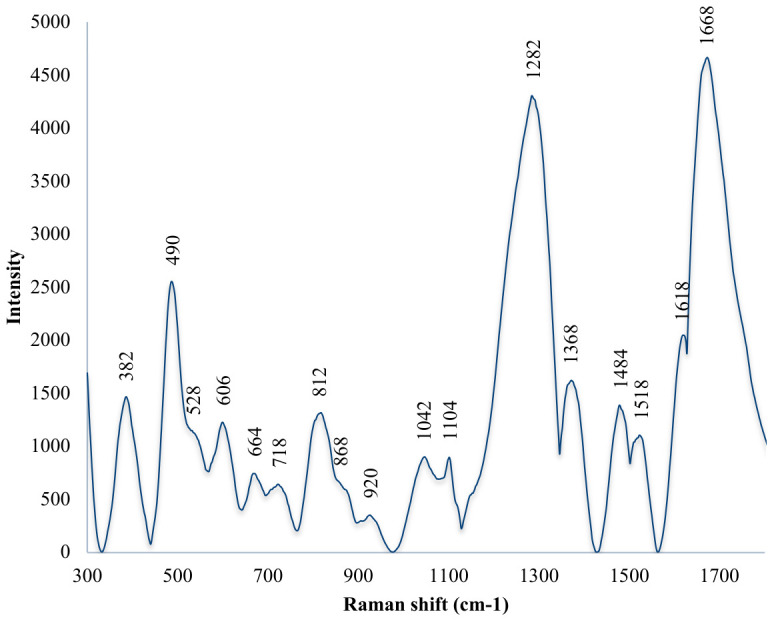
Raman spectrum of Fraction 6 of *Acinetobacter baumannii* in the range of 300–3400 cm^−1^.

**Figure A9:**
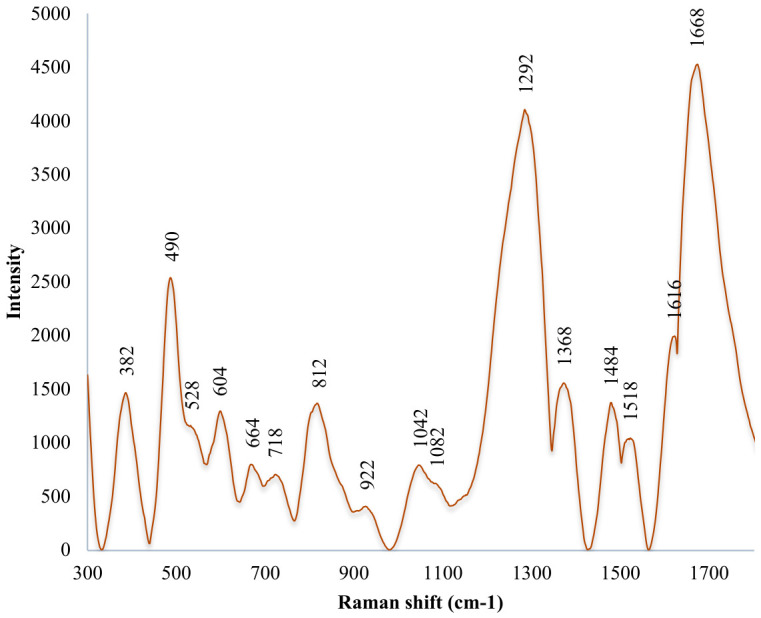
Raman spectrum of Fraction 7 of *Acinetobacter baumannii* in the range of 300–3400 cm^−1^.

Following chromatographic fractionation of *A. baumannii* the cell-free supernatant and acquisition of Raman spectra across the 300–3400 cm^−1^ range for all seven fractions (Figure A1), the cell-free supernatant (Table 1; Figure A2, Figure A3, Figure A4, Figure A5, Figure A6, Figure A7, Figure A8 and Figure A9), and the culture medium, both common and unique spectral features were identified.

Intense Raman peaks at ~1668–1670 cm^−1^, ~1518–1532 cm^−1^, ~1282–1294 cm^−1^, ~1040–1050 cm^−1^, ~528–536 cm^−1^, and ~380–392 cm^−1^ were consistently detected across all samples, including Fractions 1–7 and the cell-free supernatant (Table 1). These signals were present in all measurements and may be defined as core spectral markers of the *A. baumannii* metabolite profile.

Fraction 3 exhibited the most distinct and complex spectral profile, characterized by a series of unique or significantly altered peaks compared to other fractions and the cell-free supernatant. Characteristic signals included (Table 1): ~1724 cm^−1^, ~1450–1456 cm^−1^, ~770–806 cm^−1^, and ~446–458 cm^−1^. The peak at ~1724 cm^−1^, attributed to C=O stretching vibrations of phosphatidylserine, was detected exclusively in Fraction 3. Signals at ~1040–1050 cm^−1^, corresponding to threonine and carbohydrates, reached maximum intensity specifically in Fraction 3, whereas in other samples their intensity was significantly lower.

Notably, a series of peaks was absent exclusively in Fraction 3. Signals in the ranges ~718–720 cm^−1^, ~812–816 cm^−1^, ~868–872 cm^−1^, ~915–926 cm^−1^, ~1082–1088 cm^−1^, and ~1368–1376 cm^−1^ were detected in all fractions and the cell-free supernatant, with the exception of Fraction 3. Peaks at ~1481–1486 cm^−1^ were absent in Fractions 3 and 4, and those at ~1616–1618 cm^−1^ were absent in Fractions 3, 4, and 5. The absence of these signals may reflect the qualitative distinctiveness of the molecular composition of Fraction 3, potentially associated with selective elution or conformational specificity of the corresponding metabolites.

The spectral region ~770–806 cm^−1^ warrants particular attention. In the spectrum of the culture medium, a distinct peak at ~770 cm^−1^ was detected, whereas in Fraction 3 the signal appeared as a broadened low-intensity profile. In the cell-free supernatant and the remaining chromatographic fractions, reliable signals in this spectral region were absent, indicating fractionation of the corresponding metabolites.

Fraction 2 was characterized by unique peaks at ~990 cm^−1^ and ~998 cm^−1^, absent in all other fractions and the cell-free supernatant. Fraction 4 was distinguished by a unique signal at ~1154 cm^−1^, characteristic of bacterial carotenoids, as well as peaks in the ~1464–1468 cm^−1^ region. Fraction 6 exhibited a unique peak at ~1104 cm^−1^, attributed to phenylalanine. Fraction 7 demonstrated maximum signal intensity in the regions ~484–494 cm^−1^, ~596–606 cm^−1^, ~664–668 cm^−1^, and ~718–720 cm^−1^.

For correct interpretation of the bacterial fraction spectra, the spectrum of the original culture medium was recorded, in which peaks at ~1682, ~1608, ~1532, ~1470, ~1446, ~1378, ~1320, ~1282, ~1110, ~1080, ~1048, ~1040, ~1008, ~982, ~882, ~834, ~800, ~770, ~718, ~702, ~686, ~602, ~548, ~498, and ~370 cm^−1^ were detected. Attribution of these spectral signals, including their correspondence to individual components of the culture medium, was addressed in our previous study dedicated to the combined approach of chromatographic fractionation and Raman spectroscopy for metabolite profiling of *Enterobacter* spp. supernatant [35]. The cited publication provides a detailed interpretation of the culture medium peaks, allowing the present work to focus on the identification of signals unambiguously associated with bacterial metabolites. Additionally, in this work, powdered and crystalline myo-inositol were analyzed by Raman spectroscopy, revealing characteristic bands at: ~1357, ~1325, ~1279, ~1119, ~1063, ~1008, ~932, ~897, ~734, ~612, ~503, ~462, ~428, ~388, and ~306 cm^−1^ [35].

Thus, chromatographic fractionation combined with Raman spectroscopy enabled the identification of Fraction 3 as the most spectrally distinct, characterized by a unique pattern of absence of several amino acid and nucleotide signals alongside the presence of specific markers (in particular, phosphatidylserine). The revealed fraction-specific features open perspectives for the targeted identification of biologically active components of the *A. baumannii* secretome.

## 3. Discussion

Our data demonstrate that the combination of chromatographic fractionation and Raman spectroscopy enables effective deconvolution of the complex secretome of *A. baumannii* and facilitates the identification of fractions with unique metabolic profiles.

In the low-frequency region of the Raman spectra, a series of signals was observed, the distribution of which varied depending on the chromatographic fraction. In the range of 380–392 cm^−1^, signals attributed to deformation vibrations of C–S=O bonds in sulfoxide groups were observed in all experimental samples [12]. Peaks in the narrow range of 382–384 cm^−1^ were detected in the cell-free supernatant and most chromatographic fractions, with the exception of Fraction 3. The spectrum of this fraction in the specified region was characterized by a broad low-intensity signal, inferior in intensity to the signal of the culture medium (~370 cm^−1^), which may indicate a specific molecular composition of Fraction 3, potentially due to conformational changes of sulfur-containing compounds or their reduced concentration.

In the range of 446–458 cm^−1^, signals were registered in Fractions 3, 4, and 5, with their intensity in Fraction 3 being noticeably lower than in Fractions 4 and 5. At present, there are no unambiguous data in the available scientific literature allowing the attribution of these signals to specific metabolites of *A. baumannii*, which necessitates further research using chromatography-mass spectrometry or isotopic labeling methods.

In the spectral region of 484–494 cm^−1^, the registered signals reflect a combined contribution of several classes of biomolecules. According to Nanda et al. (2016), a peak in the region of ~490 cm^−1^ can be attributed to stretching vibrations of disulfide bonds (S–S stretching) of cysteine in protein structures, acting as a sensitive marker of the conformational state of cysteine-containing peptides and proteins [13]. Conversely, according to a review by Pezzotti (2021), the signal at ~486 cm^−1^ is attributed to C–C–O rocking/deformation vibrations in glycerol-containing lipid structures; the authors emphasize that this signal is most informative when considered in conjunction with the peak at ~675 cm^−1^, which allows this combination to be used as a specific marker of glycerol-containing lipids [14]. Additionally, in a study by Daood et al. (2020), a peak in the region of ~484 cm^−1^ was interpreted as a polysaccharide marker associated with symmetric vibrations of glycosidic bonds and deformations of cyclic structures of exopolysaccharides of the bacterial biofilm matrix [15]. Analysis of the signal intensity distribution in the region of 484–494 cm^−1^ revealed maximum intensity in Fraction 7 and a significant decrease in Fraction 3. Such spectral behavior may indicate a qualitative distinctiveness of the molecular composition of Fraction 3, potentially associated with a low content of cysteine-enriched proteins, glycerol-containing lipid components, or polysaccharides, or with a change in the conformational state of disulfide or glycosidic bonds, making the corresponding vibrational modes less accessible for detection. In the context of *A. baumannii* secretome analysis, this may reflect specific selection of soluble effectors, alternative mechanisms of protein structure stabilization, or a specialized metabolic response not requiring the accumulation of these components.

In the range of 528–536 cm^−1^, also present in all experimental samples, a different pattern of signal distribution is observed. According to Guicheteau et al. (2006), a peak in the region of ~529 cm^−1^ can be attributed to stretching vibrations of the S–S bond of cysteine or deformation vibrations of the O=C–N skeleton of asparagine and glutamine, whereas signals at 532, 534, and 536 cm^−1^ correspond to deformation modes of O=C–O and C–C–O/O=CN of alanine, leucine, and isoleucine, respectively [16]. The observed distribution of intensities—dominance of the ~528 cm^−1^ signal in the cell-free supernatant and Fractions 1, 2, 6, and 7, decreased intensity in Fractions 4 and 5 (peak ~534 cm^−1^), and minimal expression in Fraction 3 (~536 cm^−1^)—reflects differential distribution of sulfur-containing and aliphatic amino acid residues between molecular fractions. In particular, the prevalence of the signal associated with cysteine and amide groups in the extreme fractions may indicate the participation of corresponding metabolites in the formation of both high-molecular-weight protein complexes and low-molecular-weight secretory products. The decrease in intensity of signals characteristic of aliphatic amino acids in intermediate fractions may indicate their predominant intracellular localization or elution specificity.

In the spectral interval of 596–606 cm^−1^, according to the review by Pezzotti (2021), there is a minimal contribution from most membrane lipids, which increases its diagnostic value for detecting nucleotide and protein metabolites [14]. This work indicates that pyrimidine bases cytosine and uracil exhibit an intense band of C=O deformation vibrations at ~600 cm^−1^, and for uracil, this signal is dominant in the range of 600–1200 cm^−1^, which can serve as a potential marker for identifying pyrimidine-containing compounds [14]. However, analysis of the spectral atlas of amino acids and nucleotides presented by Guicheteau et al. (2006) shows that signals from nucleoside triphosphates (ATP, GTP, TTP, UTP) in the low-frequency region manifest primarily at other wavenumber values: pyridine rings of adenosine and uridine demonstrate characteristic modes at 729–732 cm^−1^ and 731 cm^−1^, respectively, whereas the imidazole ring of guanosine is registered at 652–684 cm^−1^ [16]. Direct data on a peak at ~600 cm^−1^, unambiguously attributed to deformation vibrations of the C=O of the pyrimidine ring, are not presented in the reference spectra of nucleotides cited by Guicheteau et al. (2006), which indicates the need for additional verification of the interpretation considering methodological differences [16]. In the close spectral region, signals from aromatic amino acids may contribute: according to Guicheteau et al. (2006), phenylalanine demonstrates a band of deformation of the monosubstituted benzene ring at 620 cm^−1^, whereas tyrosine exhibits modes of para-disubstituted benzene at 642 and 676 cm^−1^ [16]. In addition, vibrations of sulfur-containing amino acids are registered in the interval of 600–720 cm^−1^: for methionine, bands are characteristic at 655 cm^−1^ (CH_2_–S), 680 cm^−1^ (C–S), and 719 cm^−1^ (S–CH_3_), and for cysteine at 536 cm^−1^ (S–S), 639 and 692 cm^−1^ (C–S) [16]. Additionally, the potential contribution of lipid components should be considered. According to Pezzotti (2021), phosphatidylinositol (PI) demonstrates characteristic bands associated with C–OH bending vibrations of the inositol residue at 576 and 601 cm^−1^ [14]. The specified band at 601 cm^−1^ falls within the registered experimental range of 596–606 cm^−1^ and theoretically may contribute to the observed signals in the presence of inositol-containing metabolites in the cell-free supernatant. In addition, cholesterol exhibits low-frequency bands of highest intensity at 604 and 702 cm^−1^, attributed to deformation vibrations of the steroid ring [14]. The peak at 604 cm^−1^ is also localized within the analyzed spectral window. However, as noted in [14], these low-frequency lipid markers are rarely observed in other membrane lipids and may serve as specific indicators only provided their resolvability against the background of signals from other biomolecules. A peak at 602 cm^−1^ is detected in the spectra of the culture medium, whereas in the cell-free supernatant and most chromatographic fractions, a signal in the region of ~596 cm^−1^ is registered with insignificant variations in the maximum position. The signal intensity varies between samples: the most pronounced weakening is characteristic of Fraction 3, whereas Fraction 7 demonstrates maximum intensity, which may indicate selective enrichment with target metabolites during chromatographic separation. The observed spectral behavior may reflect the differential distribution of nucleotide-containing compounds (including pyrimidine bases with characteristic C=O bending at ~600 cm^−1^ [14]), aromatic amino acids, sulfur-containing metabolites, and/or inositol-containing lipid components between chromatographic fractions. The decrease in signal intensity in Fraction 3, in particular, may indicate a qualitative distinctiveness of its molecular composition—for example, relative depletion of certain classes of metabolites or specific selection during chromatographic separation. Considering the potential overlap of signals from various classes of compounds in the region of 596–606 cm^−1^ and the limited availability of reference data for unambiguous attribution, additional research using chromatography–mass spectrometric profiling of corresponding fractions combined with extended spectral analysis of reference compounds under identical registration conditions is advisable to verify the molecular nature of the observed signals.

In the adjacent spectral interval of 664–668 cm^−1^, signals attributed to COO^−^ wagging vibrations of the carboxylate group of valine were registered [16]. In a broader context, the range of 640–664 cm^−1^ is characteristic of deformation vibrations of carboxylate groups of aliphatic amino acids, making signals in this area sensitive markers of the presence of free amino acids or peptide fragments with exposed terminal carboxyl groups. With generally low signal intensity in all samples, Fraction 3 demonstrates minimal intensity with broadening and shifting of the maximum to the region of ~668 cm^−1^, whereas Fraction 7 is distinguished by the most pronounced intensity in this spectral region. The observed distribution reflects differential distribution of amino acid residues with exposed carboxylate groups: enrichment of Fraction 7 with low-molecular-weight peptides or free amino acids occurs, whereas broadening and shifting of the maximum in Fraction 3 are likely due to changes in microenvironment, solvation, or conformational mobility of corresponding compounds.

In the spectrum of the cell-free supernatant of *A. baumannii*, a signal in the region of 702–704 cm^−1^ was additionally detected, which appears as a shoulder of the main peak. According to Guicheteau et al. (2006), the peak at 705 cm^−1^ is attributed to C–OH twisting deformation vibrations of threonine [16]. It is important to note that this mode should be distinguished from COO^−^ wagging vibrations, which for aliphatic amino acids, including threonine, are localized in the range of 640–664 cm^−1^ [16]. The presence of an intense signal specifically in the region of ~700 cm^−1^ indicates a specific contribution of threonine hydroxyl groups to the spectral profile of the studied sample. Comparative analysis with the spectrum of the culture medium revealed the presence of a peak in the same spectral region (~702 cm^−1^); however, its intensity in the cell-free supernatant significantly exceeds the background signal of the medium. This increase in intensity indicates accumulation of threonine-containing metabolites or peptides during cultivation. Notably, no pronounced signals are registered in the analyzed spectra in the interval of 731–741 cm^−1^, which in the work of Guicheteau et al. is also attributed to C–OH twisting deformations (for serine and threonine) [16]. The absence of peaks in the region of 731–741 cm^−1^ in the presence of an intense shoulder at ~702 cm^−1^ confirms that the observed signal is due specifically to the characteristic vibrational mode of threonine at 705 cm^−1^, and not to overlapping contributions of other alcohol groups or conformational isomers. The absence of similar peaks in chromatographic fractions may indicate their loss or transformation during separation, or low concentration in individual eluates.

In the region of 718–720 cm^−1^, signals were registered in all fractions, with the exception of Fraction 3, with maximum intensity observed in Fraction 7. Interpretation of these signals is possible based on reference data on vibrational modes of amino acids and phospholipids. According to Guicheteau et al. (2006), the peak at 719 cm^−1^ is attributed to S–CH_3_ stretching vibrations of methionine [16]. At the same time, according to the review by Pezzotti (2021), the signal in the region of 718–719 cm^−1^ can be attributed to symmetric stretching vibrations of the C–N bond in the choline headgroup N^+^(CH_3_)_3_ of phosphatidylcholine and sphingomyelin [14]. The observed distribution of intensities may reflect selective distribution of methionine-containing compounds and/or phospholipids with choline headgroups between molecular fractions during chromatographic separation. The absence of a signal in the cell-free supernatant may indicate masking of corresponding vibrational modes due to intermolecular interactions in the complex matrix of the native sample or low concentration of free methionine-containing metabolites. The absence of a signal in Fraction 3, on the contrary, indicates a qualitative distinctiveness of its molecular composition—potentially, relative depletion of methionine-containing peptides or phospholipids, or specificity of elution of these compounds at other stages of chromatographic separation. The pronounced signal intensity in Fraction 7, on the contrary, indicates potential enrichment of this fraction with low-molecular-weight compounds containing methionine residues with exposed methyl groups, or phospholipids with choline headgroups.

In the spectral interval of 770–806 cm^−1^, signals were registered in the spectra of the culture medium and Fraction 3: a distinct peak at ~770 cm^−1^ is detected in the medium, whereas in Fraction 3 the signal appears as a broad low-intensity profile, the intensity of which is significantly lower than the background; in the cell-free supernatant and the remaining chromatographic fractions, reliable signals in this spectral region are absent. According to Guicheteau et al. (2006), the peak at ~773 cm^−1^ is attributed to CH_2_/CH_3_ rocking deformation vibrations and skeletal C–C stretching vibrations of cysteine [16]. In the review by Pezzotti (2021), it is indicated that a peak at ~777 cm^−1^ is characteristic of phosphatidylinositol (inositol ring vibrations), and a signal at ~783 cm^−1^ corresponds to symmetric stretching of phosphodiester bonds in DNA [14]. The observed spectral dynamics indicates qualitative heterogeneity of Fraction 3: broadening and decrease in signal intensity relative to the medium may reflect the presence of cysteine-containing peptides or nucleotide fragments in an altered solvation shell or modulation of vibrational modes by intermolecular interactions. Selective detection of the signal exclusively in Fraction 3 indicates fractionation of corresponding metabolites during chromatographic separation.

In all experimental samples, with the exception of Fraction 3, peaks in the region of 812–816 cm^−1^ were detected with intensity exceeding the background level of the culture medium, in which a signal at ~800 cm^−1^ is registered. At the same time, a systematic gradient of the maximum position is observed (816 cm^−1^ in supernatant → 814 cm^−1^ in Fractions 1, 2, 4, 5 → 812 cm^−1^ in Fractions 6, 7) and variation in intensity (maximum in Fraction 7, minimum in Fractions 4 and 5). According to Verma et al. (2020), the peak at ~812 cm^−1^ is attributed to vibrations of the phosphodiester backbone of RNA/DNA and is used as a marker of nucleic acids in bacterial cells [17]. In the review by Pezzotti (2021), it is indicated that a signal in the interval of 805–814 cm^−1^ arises from symmetric stretching vibration of phosphodiester bonds −O−P−O− in the RNA backbone, while the intensity of the peak at ~814 cm^−1^ is directly proportional to the number of nucleotide units involved in secondary interactions [14]. Additionally, according to Guicheteau et al. (2006), bands at ~816 and ~865 cm^−1^ in the interval of 800–900 cm^−1^ are characterized by pronounced spectral dominance in spectra of mono- and diphosphates relative to triphosphates, which may reflect the degree of phosphorylation of nucleotide metabolites [16]. In the study by Oliveira et al. (2021), the peak at 813 cm^−1^ was also highlighted as statistically significant for differentiating bacterial species and may be associated with the presence of tyramine in *Enterococcus* spp. or tyrosine-containing protein structures, such as protein A in *S. aureus* [18]. In the work by McEwen et al. (2010), the peak at 814 cm^−1^ was attributed to stretching vibrations of the ν(C–N) bond in the composition of extracellular polymeric substances (EPS) of Gram-negative bacteria, including *Pseudomonas putida* [19]. According to Ma et al. (2026), a peak in the interval of 815–820 cm^−1^ can be attributed to O–P–O stretching vibrations in tyrosine-containing structures or phosphodiester groups of the spore cortex, while the shift of the peak to the right reflects changes in hydrogen bonds and local hydrophobicity of the microenvironment [20].

The observed spectral dynamics indicates fractionation of nucleotide metabolites, peptide fragments, or phospholipid components with exposed phosphodiester and amino groups during chromatographic separation: the shift of the maximum to the low-frequency region (816 → 812 cm^−1^) may reflect changes in molecular environment, degree of solvation, conformational mobility, or degree of phosphorylation of corresponding functional groups. Increased signal intensity in Fraction 7 indicates its potential enrichment with nucleic acids, short peptides, components of extracellular polymer matrices, or phospholipid fragments, whereas reduced intensity in Fractions 4 and 5 indicates relative depletion of these components.

In the spectral range of 868–872 cm^−1^, pronounced signals are detected in all studied samples, with the exception of Fraction 3, with maximum intensity observed in the cell-free supernatant and Fraction 7, and minimum—in Fraction 5. According to Guicheteau et al. (2006), bands in this region correspond to stretching vibrations of C–C bonds in the polypeptide backbone and vibrations of C–COOH carboxyl groups of amino acid side chains: signals at 915–919 cm^−1^ are characteristic of valine, glycine, alanine, serine, and phenylalanine, the peak at ~916 cm^−1^ for glutamic acid, at 920–922 cm^−1^ for proline, leucine, and isoleucine, and at 925–926 cm^−1^ for alanine and glutamine [16]. Additionally, according to the review by Pezzotti (2021), the signal at ~922 cm^−1^ can be attributed to stretching vibrations of the C–COO^−^ bond of phenylalanine [14]. In the work by Saraeva et al. (2023), it was shown that skeletal stretching vibrations of COO^−^ and C–C in protein structures manifest in the interval of 918–926 cm^−1^, which confirms the predominantly protein nature of the registered signals [21]. Dominance of the peak in the region of 922 cm^−1^ in most samples indicates predominant presence of amino acids with branched aliphatic side chains (leucine, isoleucine) and/or cyclic structures (proline, phenylalanine), whereas shifting of the maximum to ~916 cm^−1^ in Fraction 4 may indicate relative enrichment with glutamic acid. Variation in intensity and position of maxima reflects selective fractionation of these protein components.

In Fraction 2 of the cell-free supernatant of *A. baumannii*, a peak in the region of ~990 cm^−1^ of low intensity was detected, unique for this fraction and absent in the spectrum of the original culture medium, where a more intense signal at ~982 cm^−1^ is observed. According to Guicheteau et al. (2006), vibrational modes in the range of 980–992 cm^−1^ are attributed to deformation vibrations of methylene groups: the peak at 986 cm^−1^ corresponds to CH_2_ wagging vibrations, and signals at 991–992 cm^−1^ as well as to methylene rocking in aliphatic fragments of amino acids [16]. The appearance of a peak at ~990 cm^−1^ exclusively in Fraction 2 may indicate selective isolation, conformational rearrangement, or change in molecular environment of aliphatic compounds during chromatographic separation. The observed shift of the maximum position (982 → 990 cm^−1^) and decrease in signal intensity in Fraction 2 compared to the culture medium may also indicate partial removal or dilution of corresponding metabolites.

Additionally, another unique peak in the region of ~998 cm^−1^ was detected in Fraction 2, the intensity of which is lower compared to the peak of the nutrient medium in the region of ~1008 cm^−1^. According to Guicheteau et al. (2006), vibrational modes in the range of 991–999 cm^−1^ are attributed to rocking vibrations of the methylene group: the peak at 999 cm^−1^ is characteristic of glutamine, and at 991 cm^−1^ for asparagine [16]. The observed peak in Fraction 2 at ~998 cm^−1^ may be associated with the presence of glutamine- or asparagine-containing peptides. In the nearby spectral region (~1004–1007 cm^−1^), peaks are attributed to stretching vibrations of the C–C skeleton of the aromatic ring of phenylalanine and tyrosine [18]. At the same time, the signal at 1004 cm^−1^ is used as a characteristic marker of phenylalanine, and the peak at 1005 cm^−1^ may be associated with carotenoids of *S. aureus* [18,19,23]. In the work by McEwen et al. (2010), the peak at 1006 cm^−1^ was identified as ring deformation vibrations (δ ring deformation) of phenylalanine in proteins, which confirms its belonging to aromatic amino acids [19]. In the study by Kusić et al. (2015), dedicated to Raman spectroscopy of bacteria and biofilms, the peak at 1004 cm^−1^ was also attributed to phenylalanine and used as a marker of proteins in bacterial cells [24]. It should be noted that in studies by Oliveira et al. [18,23], excitation at a wavelength of 830 nm was used, whereas Kusić et al. [24] used an excitation wavelength of 532 nm. In the present study, a laser with a wavelength of 785 nm was used, which may lead to differences in the relative intensities and positions of peaks due to resonance effects [18,23,24]. The low intensity of the signal at ~998 cm^−1^ indicates an insignificant content of these components in this fraction [18,23,24].

In all experimental samples, peaks in the region of ~1040–1050 cm^−1^ were detected, with maximum intensity registered in the cell-free supernatant and Fraction 3, and minimum—in Fraction 7. According to Guicheteau et al. (2006), the vibrational mode at 1043 cm^−1^ is attributed to stretching vibrations of C–C/C–N/C–NH_2_ bonds in the amino acid threonine [16]. The observed signals in the studied samples may be associated with the presence of threonine or peptides containing it. The shift of the maximum position to 1050 cm^−1^ in Fraction 3 may indicate a change in conformation or microenvironment of corresponding molecular fragments during chromatographic separation. The presence of peaks in the culture medium at 1040 and 1048 cm^−1^ indicates a possible contribution of medium components to the observed signals.

In the cell-free supernatant of *A. baumannii*, a peak in the region of 1088 cm^−1^ was detected. In Fractions 1, 2, 5, and 7, peaks in the region of 1082–1088 cm^−1^ were registered. The intensity of signals in the studied samples exceeds the intensity of the culture medium peak at 1080 cm^−1^. According to Guicheteau et al. (2006), the vibrational mode at 1080 cm^−1^ is attributed to stretching vibrations of C–C and C–O bonds in aspartic acid, as well as C–N/amino terminal group/NH_3_ twist vibrations in phenylalanine [16]. In bacterial spectra, vibrations of O–P–O of nucleic acids may also contribute in this spectral region [16]. According to Saraeva et al. (2023), the peak at 1085 cm^−1^ is attributed to stretching vibrations of PO_2_ in nucleic acids, as well as C–C and C–O bonds in lipids and carbohydrates; after electroporation of *E. coli* bacteria, this peak shifts to 1090 cm^−1^ with a simultaneous decrease in intensity, which indicates structural changes in nucleic acids [21]. According to Oliveira et al. (2012), the peak at 1084 cm^−1^ corresponds to deformation vibrations of COH and stretching vibrations of CCO [18,23]. According to Verma et al. (2019), the band at 1085 cm^−1^ is attributed to vibrations of lipids and mycolic acids, the intensity of which increases in the exponential growth phase of bacteria [28]. However, in a study by Saraeva et al. (2023), a confocal scanning Raman microspectrometer Confotec-350 (Sol Instruments, Minsk, Belarus) was used with an excitation wavelength of 520 nm and laser power of 0.5 mW, and in the work by Oliveira et al. (2012)—a dispersive spectrometer Lambda Solutions P1 with a diode laser of 830 nm and power of about 300 mW was used, which may lead to some differences in relative intensities and positions of peaks due to resonance effects [21,23]. The observed signals in the studied samples may be associated with the presence of aromatic amino acids (phenylalanine) and/or aspartic acid, as well as nucleic acids, lipids, and mycolic acids. Higher intensity of peaks in the cell-free supernatant and fractions compared to the culture medium indicates the accumulation of these biomolecules during bacterial growth.

In Fraction 6, a unique peak at ~1104 cm^−1^ was detected, the intensity of which exceeds the intensity of the culture medium signal in the region of ~1110 cm^−1^. According to Locke et al. (2022), the band at 1104 cm^−1^ is attributed to phenylalanine and is associated with vibrations of the aromatic ring of this amino acid in the composition of protein structures [25]. Higher peak intensity in Fraction 6 compared to the medium indicates selective accumulation of phenylalanine or phenylalanine-containing peptides in this eluate during chromatographic separation.

In the cell-free supernatant of *A. baumannii*, a small peak in the region of ~1128 cm^−1^ was detected. According to Pezzotti (2021), the band at ~1128 cm^−1^ is attributed to vibrations of the lipid backbone and skeletal C–C vibrations [3,14]. At the same time, the author notes that signals in this region (along with bands at 1060–1095 cm^−1^ for C–C skeletal stretching and 1070–1090 cm^−1^ for symmetric stretching vibrations of PO_2_^−^) are considered unsuitable for reliable differentiation between lipids and proteins due to significant overlap of spectral contributions [3]. According to Guicheteau et al. (2006), in the region of 1120–1152 cm^−1^, stretching vibrations of the C–C skeleton in amino acids are also observed, as well as modes associated with twisting/rocking of NH_2_/NH_3_^+^ groups [5,16]. Thus, the observed peak at ~1128 cm^−1^ in the cell-free supernatant of *A. baumannii* may reflect a cumulative contribution of lipid and protein components.

In Fraction 4, a unique peak in the region of ~1154 cm^−1^ was detected. According to Kushwaha et al. (2014), intense bands in the range of 1153–1159 cm^−1^ are characteristic of bacterial carotenoids and are attributed to stretching vibrations of C–C bonds of the conjugated backbone of the carotenoid structure [15,26]. In the study of psychrotrophic bacteria *Kocuria rosea* KK12, having pink-orange pigmentation, a peak at 1154 cm^−1^ was also observed, which along with bands at 1511–1530 cm^−1^ (stretching vibrations of C=C) and 1003–1010 cm^−1^ (deformation vibrations of C–CH_3_) constitutes a characteristic spectral fingerprint of carotenoids [15]. The presence of this peak exclusively in Fraction 4 may indicate selective accumulation of carotenoid-containing compounds during chromatographic separation. Carotenoids are known as protective pigments synthesized by bacteria in response to stressful environmental conditions, and their detection may indicate adaptation mechanisms of *A. baumannii* [15]. To confirm the nature of this signal, additional spectrophotometric analysis in the visible spectral region is advisable.

In all experimental samples, peaks in the region of 1282–1294 cm^−1^ are present. The most intense peak was registered in Fraction 1, while the lowest intensity is observed in Fraction 4 (comparable to the culture medium peak at 1282 cm^−1^). According to Verma et al. (2019), the band at ~1299 cm^−1^ is attributed to vibrations of lipids and mycolic acids, the intensity of which increases in the exponential growth phase of bacteria [28]. According to Locke et al. (2022), differences in the spectral region of 1294–1304 cm^−1^ are associated with deformation vibrations of CH_2_ in lipids, as well as with changes in the content of phospholipids [25]. According to Guicheteau et al. (2006), the peak at 1284 cm^−1^ is attributed to deformation vibrations of C–N–H (Amide III) of glutamine, and the peak at 1294 cm^−1^—to twisting and deformation vibrations of CH_2_ of cysteine [16]. According to Oliveira et al. (2012), obtained at an excitation wavelength of 830 nm, the peak at 1285 cm^−1^ is attributed to twisting vibrations of CH_2_, and the peak at 1293 cm^−1^—to deformation vibrations of CH_2_ of fatty acids and cytosine [23]. According to Saraeva et al. (2023), obtained at an excitation wavelength of 520 nm, the peak at ~1299 cm^−1^ is attributed to vibrations of CH_2_ in saturated lipids; after electroporation of *E. coli* bacteria, this peak shifts to 1298 cm^−1^ and narrows with simultaneous increase in intensity, which may indicate changes in the structure of membrane lipids [21]. The observed variations in the position of the peak in the range of 1282–1294 cm^−1^ in the studied samples may reflect differences in the composition of lipid components, as well as the contribution of amino acid residues of proteins and nucleic acid bases to this spectral region. Higher intensity of peaks in the cell-free supernatant and individual fractions compared to the culture medium indicates accumulation of these biomolecules during bacterial growth.

In the cell-free supernatant of *A. baumannii*, a unique peak in the region of ~1358 cm^−1^ was detected. According to Pezzotti (2021), the band at 1358 cm^−1^ is attributed to stretching vibrations of C–N bonds in the indole ring of tryptophan [14]. This peak is observed in complex with other characteristic signals of tryptophan: 761 cm^−1^ (symmetric breathing of the indole ring), 1009 cm^−1^ (breathing of the benzene ring), and 1558 cm^−1^ (stretching of C2═C3) [14]. According to Guicheteau et al. (2006), the peak at 1357 cm^−1^ is attributed to the tryptophan doublet in proteins and Fermi resonance between N–C in the pyrrole ring, whereas the signal at 1358 cm^−1^ may also correspond to deformation vibrations of CH and stretching vibrations of COO^−^ in alanine [16]. During enzymatic oxidation of tryptophan by indoleamine 2,3-dioxygenase with the formation of kynurenine and N′-formylkynurenine, this peak disappears due to the breaking of the pyrrole ring [14]. The observed signal in the studied samples may indicate the presence of tryptophan or tryptophan-containing peptides, as well as alanine in the cell-free supernatant of *A. baumannii*.

In all experimental samples, with the exception of Fraction 3, peaks in the region of 1368–1376 cm^−1^ were detected, the intensity of which varied between the studied fractions. According to literature data, the band at 1368 cm^−1^ corresponds to deformation vibrations of C–H and bending vibrations of the CH_3_ group in the pyrimidine ring of deoxythymidine triphosphate (dTTP) [14]. Guicheteau et al. (2006) in detail characterized this peak as a combination of vibrations: C–H in-plane bend, ring stretch, C6–H in-plane bend, C4–N3 stretch, pyrimidine ring–CH_3_ stretch, and pyrimidine semicircle stretch in thymidine [16]. The peak at 1370 cm^−1^, observed in uridine, is also attributed to stretching vibrations of C–H in the pyrimidine ring [16]. The intensity of the peak at 1368 cm^−1^ in Fractions 1, 2, 6, and 7 was at a comparable level, with maximum intensity in Fraction 6, which may indicate increased content of thymidine-containing nucleotides in this fraction.

In addition, peaks in the region of 1371–1373 cm^−1^ may be attributed to various amino acids. In particular, the peak at 1371 cm^−1^ is attributed to deformation vibrations of CH_3_ and stretching vibrations of COOH of glycine, as well as to symmetric vibrations of CO_2_^−^, deformations of Cα_2_H_2_, and stretching vibrations of C–H of serine [16]. The peak at 1373 cm^−1^ is attributed to similar vibrations of proline and glutamic acid [16]. In studies of antibiotic resistance of *E. coli*, these bands along with other nucleic acid and protein peaks demonstrated variability in intensity depending on the physiological state of cells and exposure to antibacterial drugs [17].

Shifting of the peak to the region of higher wavenumbers in Fractions 4 (1374 cm^−1^) and, 5 (1376 cm^−1^), and the culture medium (1378 cm^−1^) likely reflects changes in the molecular environment and conformation of nucleotides and amino acids [16]. Reduced signal intensity in Fractions 4 and 5 indicates decreased content of thymidine-containing compounds and/or certain amino acids, which may indicate specific distribution of these metabolites during chromatographic separation.

In the spectrum of Fraction 3, a peak in the region of 1450–1456 cm^−1^ was detected, the intensity of which is practically comparable to the peak of the culture medium at 1446 cm^−1^. According to literature data, the band at 1450 cm^−1^ corresponds to deformation vibrations of CH_2_ groups and is predominantly associated with lipids of cell membranes [14]. Raman activity in the range of 1420–1452 cm^−1^ is due to scissoring vibrations of C–H bonds and is common to all lipids, as well as proteins [14]. This peak represents a cumulative signal from phospholipids, including phosphatidylcholine, phosphatidylethanolamine, phosphatidylserine, and phosphatidylinositol, and is widely used for mapping the lipid component in biological samples [14]. According to Guicheteau et al. (2006), peaks at 1452 and 1456 cm^−1^ are attributed to deformation vibrations of CH and CH_3_ groups in various amino acids, including valine, threonine, and leucine [16]. Comparable intensity of the peak in Fraction 3 and the culture medium indicates that the content of lipid components and/or amino acids in this fraction does not exceed the background level of the medium. This may indicate low content of membrane phospholipids, their fragments, or certain amino acids in the studied fraction, which is consistent with data on the distribution of other biomolecules in chromatographic fractions.

In the cell-free supernatant of *A. baumannii* and Fraction 4, unique peaks in the region of 1464–1468 cm^−1^ were detected. In the culture medium, a peak at 1470 cm^−1^ was registered. The intensity of the peak of the cell-free supernatant exceeds the intensity of the peak of Fraction 4 in the region of 1464–1468 cm^−1^, while the intensity of the peak of Fraction 4 is comparable to the intensity of the medium peak at 1470 cm^−1^.

According to literature data, the band at 1465 cm^−1^ may be attributed to deformation vibrations (bending) of CH_2_ groups [14]. In studies of neurogenic differentiation of PC12 cells, the peak at 1464 cm^−1^ was attributed to scissoring vibrations of CH_2_ in dopamine/dopamine quinone [14]. This band is in the spectral region characteristic of deformation vibrations of methylene groups in lipids and proteins and may serve as a marker of the presence of certain classes of biomolecules.

In studies of *E. coli*, *B. cereus*, *S. aureus*, and *Salmonella* spp., the peak in the range of 1440–1475 cm^−1^ was also attributed to deformation vibrations of CH_2_ groups and is present in spectra of all studied bacteria in both liquid and solid cultures [29]. This range is characterized by high signal intensity and is an important marker for identification of microorganisms by Raman spectroscopy.

According to Guicheteau et al. (2006), the peak at 1467 cm^−1^ is attributed to deformation vibrations of CH and CH_3_ groups in glycine and serine, and the peak at 1470 cm^−1^—to similar vibrations in glutamic acid [16]. This allows suggesting that the observed peaks in the region of 1464–1468 cm^−1^ in the cell-free supernatant may be associated with the presence of amino acids, in particular glycine and serine.

Higher intensity of the peak in the cell-free supernatant compared to Fraction 4 indicates increased content of compounds containing CH_2_ groups (lipids, proteins, or amino acids) in the cell-free supernatant. Comparable intensity of the peak of Fraction 4 with the peak of the culture medium may indicate that this fraction contains predominantly medium components with minimal contribution of bacterial metabolites.

In all studied fractions, with the exception of Fractions 3 and 4, peaks in the region of 1481–1486 cm^−1^ were detected, the intensity and position of which varied between samples. According to literature data, the band at 1481 cm^−1^ may be attributed to stretching vibrations of C–N bonds in deoxyadenosine triphosphate (dATP) and represents a relatively strong signal located on the low-wave side of the band at ~1500 cm^−1^ [14]. This peak is in the spectral region free from lipid signals, making it a specific marker of nucleotides.

The peak at 1484 cm^−1^ is attributed to symmetric deformation vibrations of the NH_3_^+^ group in amino acids, in particular in tryptophan [14]. Tryptophan plays an important role in cell physiology, including protein synthesis and generation of molecules for various immunological functions [14]. In studies of *E. coli* growth, the peak at 1484 cm^−1^ was identified as a marker of nucleic acids, the intensity of which decreases as bacterial growth progresses, reflecting a change in the ratio of nucleic acids and proteins in the cell [30].

The peak at 1486 cm^−1^ is associated with differences in the structure of DNA/RNA and may serve as a marker of nucleic acids in bacterial cells [14].

The observed variations in the position and intensity of peaks in the region of 1481–1486 cm^−1^ between the studied fractions may reflect differences in the content of nucleotides (in particular, dATP), amino acids (tryptophan), and nucleic acids, which is consistent with data on the biochemical composition of the studied samples.

In all experimental samples, peaks in the region of 1518–1532 cm^−1^ were detected, which, according to literature data, correspond to stretching vibrations of the C=C (ν_1_) type of the polyene chain of carotenoids [31,32]. Variation in the position of the peak maximum in different samples may indicate the presence of structurally different carotenoid compounds or differences in the molecular environment of pigments. As Paret et al. note [27], the position of the ν_1_ band is sensitive to the length of the conjugated chain of the carotenoid and may serve as a marker for identifying specific pigments. In particular, the shift of the band to the region of 1529–1531 cm^−1^ is characteristic of xanthomonadin—a pigment associated with the membrane of *Xanthomonas* cells [27,31].

The highest signal intensity registered in Fraction 3 indicates enrichment of this fraction with carotenoid-containing components, whereas minimum intensity in Fraction 4 may reflect low content of pigments or their absence in this eluted population of molecules. It is important to note that carotenoids are strong scatterers in Raman spectroscopy, and variations in their contribution to the spectrum may significantly affect data reproducibility [32]. In this regard, to increase the reliability of spectral analysis, it is advisable to apply algorithms for correction of spectral variability, such as Extended Multiplicative Signal Correction with Subtraction of Spectral Interferents (EMSC-SIS), allowing minimization of non-specific contribution of photobleaching pigments [19].

Thus, the detected spectral features confirm the presence of carotenoid pigments in the studied bacterial supernatant and demonstrate the potential of Raman spectroscopy for differentiation of the biochemical composition of chromatographic fractions at the molecular level.

In all experimental samples, with the exception of Fractions 3–5, peaks in the region of 1616–1618 cm^−1^ were detected. The peak in the region of 1617 cm^−1^, registered in the spectra of glycine, serine, and glutamic acid, was attributed to deformation vibrations of the amino group (NH_2_ scissors/NH_2_ deformation) and the Amide II band [16]. This band reflects the contribution of peptide bonds and side radicals containing primary amino groups, which is consistent with data on the sensitivity of amide bands to conformational changes of protein structures [33]. In the context of bacterial spectra, the presence of a signal in this area may indicate high content of unpolymerized amino acids or fragments of peptidoglycan of the cell wall.

The peak at 1618 cm^−1^, characteristic of tryptophan and interpreted as aromatic ring vibration (ring vibration/ring stretch benzene derivative) [16], deserves special attention.

Thus, differential registration of bands 1617 and 1618 cm^−1^ allows us to not only clarify the amino acid composition of bacterial samples but also to obtain information on the secondary structure of proteins and organization of the cell wall, which expands the capabilities of Raman spectroscopy for the rapid identification of pathogenic microorganisms.

During spectral analysis of the cell-free supernatant and chromatographic fractions, a stable peak in the region of 1668–1670 cm^−1^ was detected, which, according to literature data, corresponds to the Amide I band and is due to stretching vibrations of C=O of the peptide bond of the protein backbone [23]. The intensity of this signal varied depending on the analyzed sample: maximum values were registered for the cell-free supernatant, as well as Fractions 1, 2, 6, and 7; moderately reduced intensity was observed in Fraction 5, and even lower in Fraction 3, whereas in Fraction 4 the signal reached the level of the control medium.

Such distribution of amide band intensity may reflect the heterogeneity of protein composition of the studied samples and the efficiency of their separation during chromatography.

Comparison of the obtained data with the results of studies by Kusić et al. [24], dedicated to Raman spectroscopy of bacteria under nutrient deficiency conditions, allows suggesting that variability of Amide band intensity may be associated with the physiological state of the bacterial culture. The authors showed that decrease in metabolic activity, reflected in reduction of protein content (bands 1669 and 1238 cm^−1^), is characteristic of cells adapted to poor media and may correlate with transition to a viable but non-culturable state (VBNC) [24]. In our study, the profile of protein signal distribution across fractions reflects the result of chromatographic separation of molecules with different physicochemical properties, which leads to selective enrichment or depletion of individual eluates with certain protein components.

A unique peak at 1724 cm^−1^, detected in Fraction 3, may be attributed to stretching vibrations of the C=O bond of phosphatidylserine. According to Pezzotti (2021), in Raman scattering spectra of phosphatidylserine, characteristic absorption is observed in the region of 1723 cm^−1^, corresponding to stretching of the carbonyl group [14]. Notably, in the pure compound, phosphatidylserine manifests as a doublet at 1716 and 1732 cm^−1^, however, in biological samples these components merge into a singlet peak with a center at an intermediate wavenumber of 1723 cm^−1^ [14]. The presence of this peak exclusively in Fraction 3 indicates specific distribution of phosphatidylserine between the studied fractions. Phosphatidylserine is an important component of cell membranes and plays a key role in various cellular processes, including apoptosis and signal transduction [34]. Its detection in a specific fraction may indicate the presence of membrane vesicles or fragments of cell membranes enriched with this phospholipid.

Thus, detection of the Amide I band in various fractions of the cell-free supernatant confirms the presence of protein components in the studied samples and demonstrates the potential of combining chromatographic separation with Raman spectroscopy for differential analysis of secretory profiles of bacterial cultures. Further identification of specific protein targets in the most intense fractions is advisable using methods such as mass spectrometry or immunoblotting.

## 4. Materials and Methods

### 4.1. Bacterial Strain and Culture Conditions

We used *Acinetobacter baumannii* strain ATCC 1896. Bacteria were cultured on agar plates under appropriate biosafety containment, in accordance with institutional protocols for handling pathogenic microorganisms.

### 4.2. Preparation of Conditioned Media from ESKAPE Bacteria

To prepare conditioned media, bacterial cultures were inoculated into α-MEM medium (Biolot, Saint-Petersburg, Russia (Item 1.3.9.2.)) supplemented with 0.2 mM myo-inositol (Sigma, St. Louis, MO, USA (Product of Chine) (I7508-50G)), 0.02 mM folic acid (Sigma, USA (Product of Chine) (F7876)), and 2 mM L-glutamine (Sigma, USA (Product of Chine) (G8540)) to a final concentration of 1 × 10^8^ CFU/mL. Cultivation was carried out in sterile glass vials containing 5 mL of medium for 24 h at 37 °C under 5% CO_2_ atmosphere. Following incubation, the supernatant was filtered through 0.45-μm pore-size syringe filters (Sarstedt, Nümbrecht, Germany) to remove bacterial cells.

### 4.3. Size Exclusion Chromatography (SEC)

Cell-free bacterial supernatants were concentrated using a CentriVap Vacuum Concentrator (Labconco Corp., Kansas City, MI, USA) to a volume yielding a total protein concentration exceeding 5 mg/mL. Fractionation was performed using an Agilent 1260 Infinity II high-performance liquid chromatography system equipped with OpenLAB CDS ChemStation software (Workstation version A.02.02) (Agilent Technologies, Inc., Santa Clara, CA, USA) (Figure A1). Separation was carried out under non-denaturing conditions on an Agilent Bio SEC-3 size-exclusion column (3 μm, 300 Å, 4.6 × 300 mm; Agilent Technologies, Inc., Santa Clara, CA, USA), with bi-distilled water as the mobile phase (pH ~ 7.0, ionic strength ≈ 0). The analysis was conducted in isocratic mode at 4 °C and a flow rate of 0.35 mL/min. A diode array detector monitored absorbance at 210, 230, 254, and 280 nm over a 30-min run. Due to the continuous elution profile, fractions were collected in 3-min intervals starting from minute 3. No protein was detected after minute 24, and this late-eluting material was excluded from further analysis. The collected fractions were reconcentrated to 2.5 mL using the CentriVap Vacuum Concentrator to standardize sample volume, as protein concentrations were below the detection limit. Fractions were then filtered through 0.45-μm syringe filters (Corning Inc., Corning, New York, NY, USA), frozen at −80 °C, and stored for up to two weeks prior to analysis.

### 4.4. Raman Spectroscopy

Raman spectra were acquired using an OPTEC 785LRam spectrometer (OPTEC, Saint-Petersburg, Russia) equipped with a 785-nm laser (power adjustable between 10 and 300 mW). For all measurements reported herein, the laser power at the sample was fixed at 300 mW, with an exposure time of 4 s and 100 accumulated scans per spectrum. The spectral range spanned 300–3400 cm^−1^ with a resolution of 8 cm^−1^. Samples were analyzed in the liquid phase in 2-mL glass vials. At least three independent spectra were recorded per sample, each averaged over 100 scans. Prior to measurement, samples were equilibrated to room temperature and thoroughly mixed. Baseline correction was performed using iterative polynomial approximation. Spectral data were processed using BWSpec4 and OPTEC-Raman v1.2 software.

## 5. Conclusions

Comparison of the spectra of the cell-free supernatant of *A. baumannii* and its chromatographic fractions with the spectral profile of the culture medium enabled the differentiation of signals associated with bacterial metabolites. Since the intensity of medium-specific peaks was consistently lower in bacterial samples compared to the control, the observed spectral contributions are predominantly attributed to products of bacterial metabolism. Nevertheless, definitive verification of the molecular nature of these signals requires additional research using chromatography-mass spectrometry.

Two groups of Raman signals were identified in the cell-free supernatant of *A. baumannii* and its fractions: peaks originating from the culture medium (~370, ~602, ~770, ~1040, ~1048, ~1080, ~1378, ~1446, ~1470 cm^−1^), which likely reflect residual medium components that are attenuated or masked during chromatographic separation; putative markers of bacterial metabolism (absent in the pure medium spectrum)—~380–392, ~446–458, ~484–494, ~528–536, ~596–606, ~664–668, ~702–704, ~718–720, ~770–806, ~812–816, ~868–872, ~915–926, ~990, ~998, ~1040–1050, ~1082–1088, ~1104, ~1128, ~1154, ~1282–1294, ~1358, ~1368–1376, ~1450–1456, ~1464–1468, ~1481–1486, ~1518–1532, ~1616–1618, ~1668–1670, and ~1724 cm^−1^.

The combined approach of chromatographic fractionation and Raman spectroscopy successfully enabled deconvolution of the complex secretome of the Gram-negative pathogen *A. baumannii*. A key finding was the identification of Fraction 3 as uniquely distinct in its metabolite profile: it lacks markers of methionine-containing compounds and choline phospholipids (~718–720 cm^−1^), phosphodiester backbones of nucleic acids (~812–816 cm^−1^), polypeptide backbones and amino acid side chains (~868–872, ~915–926 cm^−1^), which sharply distinguishes it from all other fractions and the cell-free supernatant. This fraction demonstrates maximum signal intensity indicative of enrichment with functionally significant biomolecules—in particular, threonine and carbohydrates (~1040–1050 cm^−1^), and carotenoids (~1518–1532 cm^−1^), as well as a unique marker of phosphatidylserine (~1724 cm^−1^), which may indicate the presence of membrane vesicles or fragments of cell membranes.

A comparative analysis of spectral profiles across different fractions revealed their specific biochemical specialization: Fraction 2 is enriched with signals of methylene groups attributed to glutamine and asparagine (~990, ~998 cm^−1^); Fraction 4 is characterized by a unique marker of bacterial carotenoids (~1154 cm^−1^) and signals of CH_2_ groups of lipids/proteins (~1464–1468 cm^−1^); Fraction 6 contains a unique phenylalanine peak (~1104 cm^−1^); and Fraction 7 demonstrates maximum signal intensity of cysteine-containing proteins, nucleotides, and phospholipids (~484–494, ~596–606, ~664–668, ~718–720 cm^−1^).

In addition, variation in the position and intensity of peaks in the regions ~812–816 cm^−1^ and ~1368–1376 cm^−1^ between fractions reflects differences in microenvironment, degree of solvation, and conformational mobility of nucleotide and amino acid residues, emphasizing the heterogeneity of the *A. baumannii* secretome.

The obtained data confirm that chromatographic fractionation effectively reduces spectral complexity, allowing Raman spectroscopy to resolve unique “metabolic fingerprints” of individual fractions. Detailed characterization of the metabolite profile of *A. baumannii* is critical for deciphering the mechanisms of pathogenicity and antibiotic resistance of this pathogen. Secreted metabolites mediate key survival processes—including intercellular communication, biofilm formation, modulation of the host immune response, and mechanisms of antimicrobial resistance. Identification of specific spectral signatures—such as stress-response markers (carotenoids) or extracellular matrix components (phosphatidylserine)—allows functional assessment of bacterial population states and detection of potential virulence factors in their native conformation.

Although final molecular identification requires complementary approaches, such as mass spectrometry and enzymatic hydrolysis, notably, Raman spectroscopy has demonstrated clinical utility for rapid bacterial identification with high accuracy [36], suggesting that our SEC–Raman workflow could be similarly adapted for rapid biomarker screening in clinical settings. Furthermore, emerging sensor platforms leveraging quantum-engineered heterostructures for sub-ppb biomarker detection [37] illustrate a promising pathway for integrating SEC–Raman molecular profiling into next-generation high-sensitivity diagnostic devices. The proposed hybrid methodology thus opens new avenues for functional screening of the bacterial secretome and may be applied to the search for biomarkers associated with virulence and antibiotic resistance of *A. baumannii*.

## Figures and Tables

**Table 1:** Putative assignment of Raman shifts to vibrational modes and biomolecular classes.

Raman Shift (cm^−1^)	Putative Assignment	Detected in	References
~380–392	Deformation vibrations of C–S=O in sulfoxide groups	cell-free supernatant of *A. baumannii*, fractions 1–7	[12]
~446–458	Unidentified signals (potentially specific metabolites of *A. baumannii)*	Fractions 3–5	–
~484–494	S–S stretching of cysteine/proteins; C–C–O rocking of glycerol-lipids; polysaccharides	cell-free supernatant of *A. baumannii*, Fractions 1–7	[13,14]
~528–536	S–S bonds of cysteine; O=C–N of asparagine/glutamine; aliphatic amino acids	cell-free supernatant of *A. baumannii*, Fractions 1–7	[15,16]
~596–606	C=O deformation vibrations of pyrimidines; aromatic amino acids; inositol-containing lipids	cell-free supernatant of *A. baumannii*, Fractions 1–7	[14,16]
~664–668	COO^−^ wagging of valine; carboxylate groups of amino acids	cell-free supernatant of *A. baumannii*, Fractions 1–7	[16]
~702–704	C–OH twisting of threonine	cell-free supernatant of *A. baumannii*	[16]
~718–720	S–CH_3_ of methionine; choline headgroups of phospholipids	Fractions 1, 2, 4–7	[14,16]
~770–806	Cysteine; phosphatidylinositol; phosphodiester bonds of DNA	Fraction 3	[14,15,16]
~812–816	Phosphodiester backbone of RNA/DNA; phosphorylated nucleotides	cell-free supernatant of *A. baumannii*, Fractions 1, 2, 4–7	[14,16,17,18,19,20]
~868–872	C–C of polypeptide backbone; amino acid side chains	cell-free supernatant of *A. baumannii*, Fractions 1, 2, 6	[16]
~915–926	Stretching vibrations of C–C in polypeptide backbone of proteins; side chains of amino acids (valine, glycine, alanine, serine, phenylalanine, glutamate, proline, leucine, isoleucine); C–COO^−^ of phenylalanine	cell-free supernatant of *A. baumannii*, Fractions 1, 2, 4–7	[14,16,21,22]
~990; ~998	Methylene groups (CH_2_ wagging/rocking); glutamine/asparagine	Fraction 2	[16,18,19,23,24]
~1040–1050	Threonine (C–C/C–N/C–NH_2_); carbohydrates	cell-free supernatant of *A. baumannii*, Fractions 1–7	[16]
~1082–1088	Phenylalanine (C–N stretching/amino terminal group/NH_3_ twist); aspartic acid (C–C stretching/C–O stretching); nucleic acids; lipids (C–C)	cell-free supernatant of *A. baumannii*, Fractions 1, 2, 5, 7	[3,5,10,11,13,22]
~1104	Aromatic ring of phenylalanine	Fraction 6	[25]
~1128	Lipid backbone; skeletal C–C vibrations; C–N vibrations in proteins	cell-free supernatant of *A. baumannii*	[14,16]
~1154	Carotenoids (conjugated C–C/C=C polyene systems)	Fraction 4	[15,26,27]
~1282–1294	Amide III; CH_2_ of lipids; cysteine; cytosine	cell-free supernatant of *A. baumannii*, Fractions 1–7	[16,21,23,25,28]
~1358	Indole ring of tryptophan (C–N); alanine	cell-free supernatant of *A. baumannii*	[14,16]
~1368–1376	dTTP (pyrimidine ring); glycine, serine, proline, glutamate, nucleic acids	Fractions 1, 2, 4–7	[14,16,17,22]
~1450–1456	CH_2_ of lipids; amino acids (valine, threonine, leucine)	Fraction 3	[14,16]
~1464–1468	CH_2_ of lipids/proteins; glycine, serine, glutamate	cell-free supernatant of *A. baumannii*, Fraction 4	[14,16,29]
~1481–1486	dATP (C–N); NH_3_^+^ of tryptophan; nucleic acids	Fractions 1, 2, 5–7	[14,30]
~1518–1532	Carotenoids (conjugated C–C/C=C polyene systems)	cell-free supernatant of *A. baumannii*, Fractions 1–7	[27,31,32]
~1616–1618	Amide II/NH_2_ deformation; aromatic ring of tryptophan	cell-free supernatant of *A. baumannii*, Fractions 1, 2, 6, 7	[16,33]
~1668–1670	Amide I (C=O of peptide bond)	cell-free supernatant of *A. baumannii*, Fractions 1–7	[24]
~1724	Phosphatidylserine (putative; C=O carbonyl group); lipid ester C=O/carbonyl groups of membrane lipids	Fraction 3	[14,34]

## Data Availability

The original contributions presented in this study are included in the article. Further inquiries can be directed to the corresponding author.
